# Thrombomodulin and syndecan-1 and association with lung function in liver transplant recipients

**DOI:** 10.3389/fimmu.2026.1662030

**Published:** 2026-04-13

**Authors:** Cecilie Cohrt Nebel, Nicoline Arentoft, Paul Suno Krohn, Ask Bock, Michael Perch, Annette Dam Fialla, Jesper Bach Hansen, Jesper Rømhild Davidsen, Niels Kristian Aagaard, Allan Rasmussen, Susanne Dam Nielsen

**Affiliations:** 1 Department of Infectious Diseases, Copenhagen University Hospital – Rigshospitalet, Copenhagen, Denmark; 2 Department of Transplantation and Digestive Diseases, Copenhagen University Hospital – Rigshospitalet, Copenhagen, Denmark; 3 Department of Clinical Medicine, Faculty of Health and Medical Sciences, University of Copenhagen, Copenhagen, Denmark; 4 Department of Cardiology, Heart and Lung Transplant Unit, Copenhagen University Hospital – Rigshospitalet, Copenhagen, Denmark; 5 Department of Gastroenterology, Odense University Hospital, Odense, Denmark; 6 Department of Gastroenterology and Hepatology, Aalborg University Hospital, Aalborg, Denmark; 7 South Danish Center for Interstitial Lung Diseases (SCILS), Department of Respiratory Medicine, Odense University Hospital, Odense, Denmark; 8 Department of Hepatology and Gastroenterology, Aarhus University Hospital, Aarhus, Denmark

**Keywords:** cohort study, comorbidity, liver transplantation, lung function, markers of endothelial dysfunction, spirometry

## Abstract

**Introduction:**

Liver transplant recipients have an increased risk of pulmonary complications, yet the pathogenesis remains poorly elucidated. The pulmonary endothelium is central in maintaining lung function, and endothelial dysfunction may contribute to airflow obstruction. Soluble thrombomodulin (TM) and syndecan-1 (SDC-1) are markers of endothelial damage, but whether TM and SDC-1 are related to lung function in liver transplant recipients remain unknown. This study investigated whether TM and SDC-1 are associated with forced expiratory volume in one second (FEV_1_), forced vital capacity (FVC), and airflow obstruction in liver transplant recipients.

**Methods:**

We included liver transplant recipients from The Danish Comorbidity in Liver Transplant Recipients (DACOLT) study. Airflow obstruction was defined as FEV_1_/FVC <0.7. TM and SDC-1 were dichotomized, and an elevated concentration was defined as above the 3^rd^ quartile. Outcomes were analyzed using linear and logistic regression.

**Results:**

340 liver transplant recipients were included. Liver transplant recipients with elevated TM had 39.5 mL lower FEV_1_ than liver transplant recipients with low TM (95% CI: -173.7;94.7, p=0.564), and 72.2 mL lower FVC (95% CI: -227.2;82.8, p=0.362), adjusted for confounders. The odds ratio (OR) for airflow obstruction was 1.01 (95% CI: 0.43;2.38, p=0.984). Liver transplant recipients with elevated SDC-1 had 93.4 mL lower FEV_1_ than liver transplant recipients with low SDC-1 (95% CI: -223.1;36.3, p=0.159) and 31.2 mL lower FVC (95% CI: -181.6;119.2, p=0.684), adjusted for confounders. The OR for airflow obstruction was 0.97 (95% CI: 0.42;2.27, p=0.950).

**Conclusion:**

In this study, we found no significant associations between TM or SDC-1 and FEV_1_, FVC, and airflow obstruction in liver transplant recipients. Further research is warranted to elucidate the mechanisms underlying pulmonary complications in liver transplant recipients.

## Introduction

Liver transplantation is the only curative treatment for patients with end-stage liver disease. Despite advancements in medical care and surgical techniques that have improved the post-transplant prognosis, liver transplant recipients still face significant complications (1). Common complications are infections and rejection of the transplanted organ (2), however, pulmonary complications such as pleural effusion, atelectasis, pulmonary edema, and pneumonia may also contribute to increased morbidity and mortality post-transplantation (3). Liver transplant recipients are particularly susceptible to pulmonary infections due to lifelong immunosuppressive medication, which may have long-lasting effects on the lungs and increase the risk of chronic pulmonary disease (4, 5). Furthermore, elevated levels of fractional exhaled nitric oxide with an expiratory flow of 50 mL/s (F_E_NO_50_) in liver transplant recipients have been reported. This may further reflect ongoing eosinophilic airway inflammation (6), raising concerns about their long-term pulmonary health.

The pulmonary vascular endothelium contributes to the regulation of lung function. It regulates inflammation and vascular permeability, which are critical factors in respiratory diseases, including chronic obstructive pulmonary disease (COPD) and emphysema (7). Thus, endothelial dysfunction has been reported in patients with COPD (8), and correlates with COPD severity and airflow obstruction (9, 10). Given these associations, endothelial damage may contribute to airflow obstruction in liver transplant recipients. Thrombomodulin (TM), an endothelial glycoprotein, is involved in coagulation regulation and is released into the bloodstream during endothelial damage (11). Elevated soluble TM has been observed in patients with acute respiratory distress syndrome, COVID-19, and community-acquired pneumonia. In these conditions, it is associated with endothelial damage, disease severity, and prognosis (12). Syndecan-1 (SDC-1) is a heparan sulphate proteoglycan found on the surface of endothelial cells (11). Increased SDC-1 indicates endothelial damage and is associated with increased disease activity in COVID-19 and trauma patients (13–15), as well as respiratory failure and mortality in patients with sepsis-related pneumonia (16). Thus, the two markers of endothelial dysfunction, TM and SDC-1, have been associated with pulmonary complications. Despite the unclear pathogenesis of airflow obstruction in liver transplant recipients, their potential associations with airflow obstruction in this group have not been investigated.

This study aimed to investigate whether TM and SDC-1 were associated with FEV_1_, FVC, and airflow obstruction in liver transplant recipients. We hypothesized that elevated TM and SDC-1 were associated with lower FEV_1_, lower FVC, and with airflow obstruction.

## Methods

### Study design

**Figure 1:**
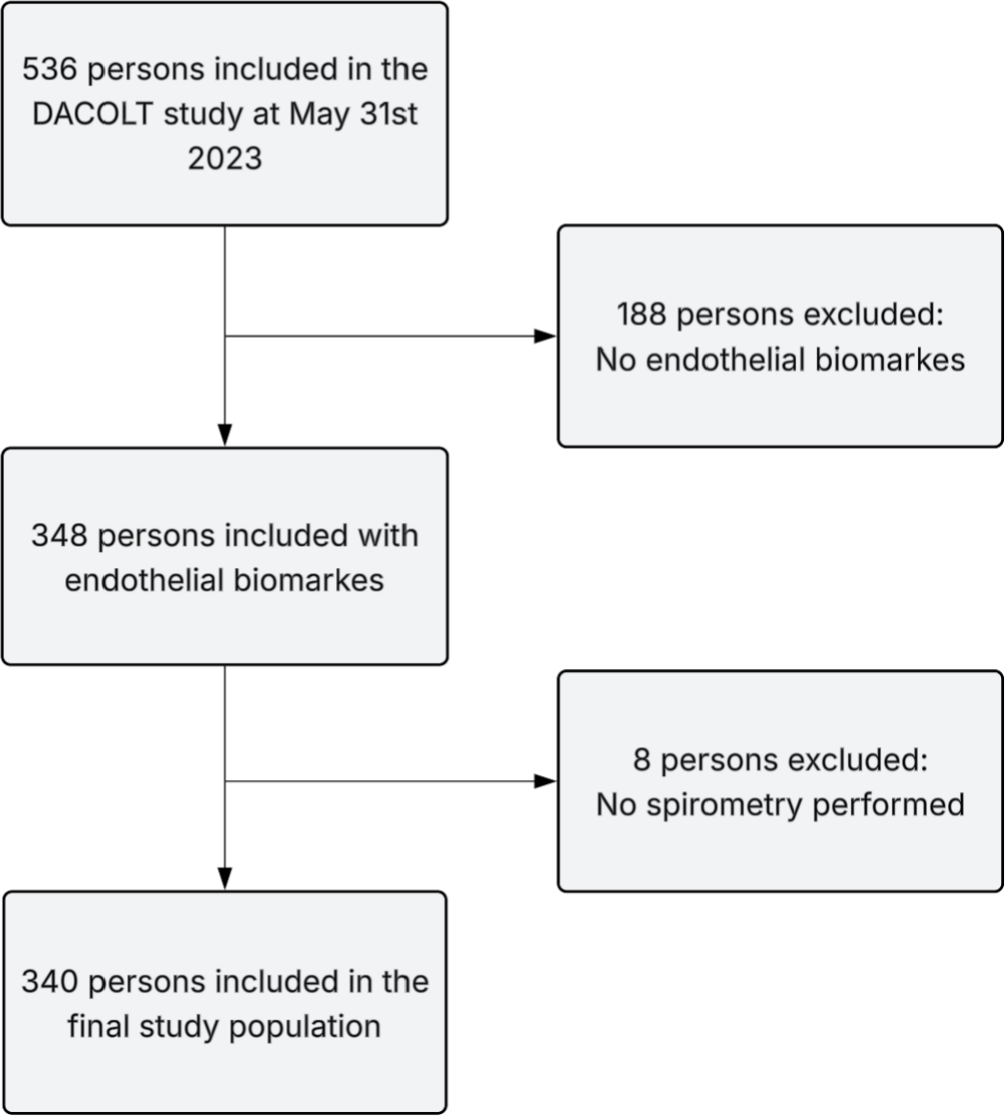
Flowchart of the participant selection process.

The Danish Comorbidity in Liver Transplant Recipients (DACOLT) study is a national, non-interventional, prospective cohort study designed to examine the burden of comorbidities in liver transplant recipients. Eligible participants include all liver transplant recipients above 20 years of age living in Denmark, who are followed at one of the four centers: Copenhagen University Hospital – Rigshospitalet, Aarhus University Hospital, Odense University Hospital, and Aalborg University Hospital, and who can provide informed consent. The cohort includes more than 80% of all liver transplant recipients in Denmark (17). This study included all participants included in the DACOLT study with available measurements of both TM and SDC-1, and FEV_1_ and FVC measured by spirometry before May 31^st^ 2023 (**Figure 1**).

The DACOLT study is approved by the Committee on Health Research Ethics of the Capital Region of Denmark (approval number H-20052199) and registered with Clinical Trials identifier NCT04777032. The study was conducted in accordance with the Declaration of Helsinki.

### Lung function

Lung function was assessed using protocolized research spirometry. Spirometry was performed by trained healthcare professionals with the EasyOne World Spirometer (ndd Medical, Zurich, Switzerland) with one-use spirettes (ndd Medical). Procedures followed the guidelines of the European Respiratory Society (ERS) (18), except participants were standing and did not wear a nose clip. Measurements included FEV_1_ and FVC. Airflow obstruction was defined as FEV_1_/FVC ratio <0.7, as recommended by the Global Initiative for Chronic Obstructive Lung Disease 2025 report (19).

### Markers of endothelial damage

TM and SDC-1 plasma concentrations were measured in venous blood samples collected from participants at time of inclusion. Samples were analyzed at the Department of Clinical Immunology, Copenhagen University Hospital – Rigshospitalet, Denmark. Analyses were performed using Luminex Human Discovery Assay (4-Plex) LXSAHM-04 in a 1:2 dilution, according to the manufacturer’s instructions. As no established clinical cut-off exists for circulating TM and SDC-1 levels, we pre-defined the upper quartile (75th percentile) as the threshold to distinguish liver transplant recipients with elevated versus low concentrations in our statistical analysis plan. This approach has been used in previous studies evaluating TM and SDC-1 as potential biomarkers of endothelial damage (20, 21). It also allows comparison of the two subgroups (elevated versus low levels of TM/SDC-1) while maintaining sufficient sample size in each category. Furthermore, this approach aims to identify a potential subgroup of participants at highest risk of airflow obstruction.

### Liver transplantation related variables

Information on liver transplantation related variables was retrieved from medical records. Immunosuppressive medication at time of inclusion was retrieved from the national Danish Shared Medication Record (FMK), which includes data on all prescriptions issued at both hospitals and general practices in Denmark. Time since transplantation was defined as the interval from the liver transplantation to study inclusion. Cirrhosis was defined according to the histological report on the liver explant. Acute rejection was defined as having one or more biopsy-verified acute rejections treated with methylprednisolone for 3–5 days. Reason for transplantation was retrieved from medical records and categorized as autoimmune liver disease, alcoholic or cryptogenic cirrhosis, fulminant hepatic failure, hepatitis C, and other. The category “other” included cancer, metabolic disease, metabolic dysfunction-associated steatohepatitis (MASH), hepatitis B, polycystic liver disease, biliary atresia, poisoning, and other rare conditions.

### Self-reported variables

Information on ethnicity and smoking history was collected from questionnaires at time of inclusion. Smoking status was classified as current, former, or never smoking. Cumulative smoking was assessed in pack years, including only current and former smokers. It was defined as the number of years an individual smoked 20 cigarettes per day. Ethnicity was determined based on self-reported country of origin of the participant’s grandparents.

### Statistical analyses

Normally distributed continuous variables and non-normally distributed continuous variables were reported as means with standard deviations (SD) and medians with interquartile ranges (IQR), respectively. Categorical variables were reported as frequencies and percentages. T-test and Fisher’s test were used to compare continuous and categorical variables, respectively.

We investigated potential associations between FEV_1_, FVC, and airflow obstruction and plasma levels of TM and SDC-1. In the primary analysis, plasma levels of TM and SDC-1 were dichotomized at the 3^rd^ quartile. Liver transplant recipients above this threshold were classified as having ‘elevated’ levels, and those below as having ‘low’ levels. As exploratory analyses, TM and SDC-1 were also analyzed as continuous variables (log2-transformed).

Statistical analyses were prespecified in a comprehensive statistical analysis plan. To investigate whether TM and SDC-1 were associated with FEV_1_ and FVC, we used multivariable linear regression. Model assumptions were tested with residual plots. All analyses were performed in a minimally adjusted model, only adjusted for age (continuous) and sex (male/female). Subsequently, a fully adjusted model was applied, which included age, sex, height (continuous), ethnicity (Caucasian/other), and smoking status (current/former/never). Based on existing literature demonstrating that lung function differs according to age, sex, height, ethnicity, and smoking status (22, 23), these variables were selected *a priori* as potential confounders. Associations between TM and SDC-1 and airflow obstruction were investigated using multivariable logistic regression analyses, adjusting for the same covariates.

We also investigated whether immunosuppressive medication, time since transplantation, or cirrhosis at time of transplantation influenced the association between TM and SDC-1 and airflow obstruction. This was done using multivariable linear and logistic regression analyses. The liver transplantation related variables were added to the linear and logistic models one at a time.

Interaction analyses between smoking status and elevated TM/SDC-1, as well as TM or SDC-1 as continuous variables, were performed for each outcome. For all outcomes, analyses were repeated stratified by smoking status (**Supplementary Tables 4** and **5**).

As an exploratory analysis, we additionally examined soluble CD163 (sCD163) as a marker of innate immune activation to explore whether innate immune pathways were associated with lung function (**Supplementary Tables 6** and **7**).

Four sensitivity analyses were performed to assess the consistency of our findings, each targeting specific potential confounders that could influence the association: Three of these, excluding liver transplant recipients transplanted with autoimmune disease, excluding liver transplant recipients with acute rejection, and excluding participants receiving prednisolone treatment, were performed to minimize the influence of immune-mediated processes, which might independently affect both endothelial biomarkers and airflow obstruction. In a fourth sensitivity analysis, only participants with cirrhosis at time of transplantation were included, as this subgroup represents individuals with a higher burden of comorbidity. All analyses were adjusted for age, sex, ethnicity, and smoking status.

To account for multiple comparisons across different outcomes, we report p-values for fully adjusted models and corresponding Benjamini–Hochberg–adjusted p-values. Furthermore, a *post hoc* power calculation was performed to evaluate the difference in prevalences of airflow obstruction between the groups with elevated and low TM/SDC-1 necessary to demonstrate a statistically significant difference. FEV_1_ and FVC were presented as differences in milliliters (mL) with a 95% confidence interval (CI). P-values ≤0.05 were considered statistically significant. All data analyses were performed in R (version 4.3.0).

## Results

### Clinical characteristics in the overall cohort

**Figure 2:**
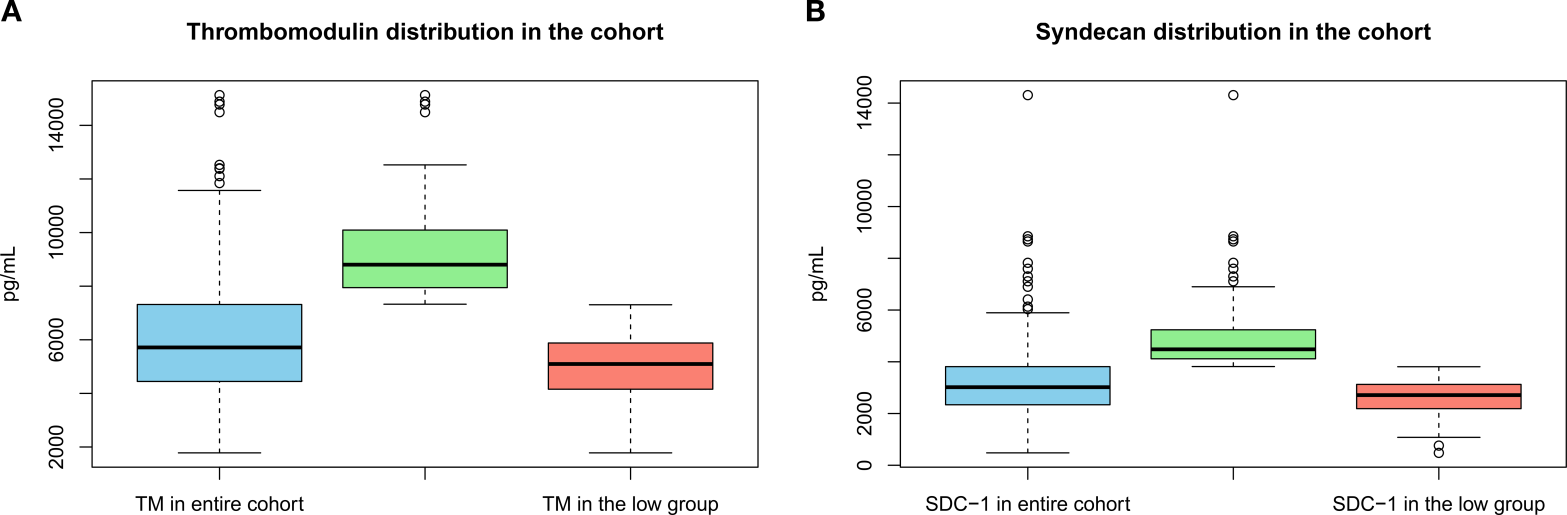
**(A, B)** Boxplots showing the distribution of thrombomodulin and syndecan-1 in the entire cohort, the elevated group, and the low group.

We included 340 liver transplant recipients in this study (**Figure 1**). The clinical characteristics were summarized in **Table 1**. Median age was 56 years, and 57% of liver transplant recipients were male. The median body mass index was 26.9 kg/m², and 50.3% were never smokers. There were no significant differences in baseline characteristics (age, sex, BMI, and smoking status) between recipients with and without spirometry. Median time since transplantation was 7.1 years. Autoimmune liver disease was the most frequent reason for transplantation (44.1%). At time of transplantation, 65.9% of the overall cohort had pathology confirmed cirrhosis. Tacrolimus and mycophenolate mofetil were the most used immunosuppressive medications, prescribed to 82.6% and 72.6% of liver transplant recipients, respectively. Median plasma concentration was 5715 pg/mL (IQR: 4451-7312) for TM and 3017 pg/mL (IQR: 2343-3810) for SDC-1. A visual presentation of TM and SDC-1 distribution can be seen in **Figures 2A, B**.

**Table 1:** Characteristics, pulmonary and liver transplantation related variables.

	Overall *n* = 340	TM below 3^rd^ quartile *n* = 255	TM above 3^rd^ quartile *n* = 85	p-value	SDC-1 below 3^rd^ quartile *n* = 255	SDC-1 above 3^rd^ quartile *n* = 85	p-value
Characteristics							
Age (years), median (IQR)	55.7 (46.8-64.6)	54.1 (44.1-63.0)	59.5 (53.6-67.8)	<0.001	54.7 (45.6-63.2)	57.7 (50.9-67.6)	0.007
Sex (male), *n* (%)	194 (57.0%)	140 (54.9%)	54 (63.5%)	0.206	138 (54.1%)	56 (65.9%)	0.059
BMI (kg/m²), median (IQR)	26.9 (24.0-30.1)	26.7 (24.1-29.9)	27.8 (24.0-30.9)	0.322	26.9 (24.0-29.9)	26.8 (23.8-30.7)	0.676
Ethnicity (Caucasian), *n* (%)	316 (92.9%)	235 (92.2%)	81 (95.3%)	0.464	233 (91.4%)	83 (97.6%)	0.052
Smoking status				0.002			0.758
- Current smokers, *n* (%)	43 (12.6%)	27 (10.6%)	16 (18.8%)		32 (12.5%)	11 (12.9%)	
- Former smokers, *n* (%)	118 (34.7%)	81 (31.8%)	37 (43.5%)		91 (35.75)	27 (31.8%)	
- Never smokers, *n* (%)	171 (50.3%)	142 (55.7%)	29 (34.1%)		125 (49.0%)	46 (54.1%)	
Cumulated smoking (pack years), median (IQR)	10.0 (4.0-20.0)	9.8 (3.2-18.0)	11.3 (5.4-27.7)	0.322	10.0 (4.0-20.0)	11.0 (5.3-27.7)	0.550
Pulmonary variables							
FEV_1_ (mL), median (IQR)	2790 (2230-3498)	2850 (2330-3555)	2500 (2060-3300)	0.004	2830 (2290-3530)	2690 (2120-3400)	0.254
FVC (mL), median (IQR)	3675 (2980-4400)	3835 (3035-4455)	3430 (2690-4190)	0.006	3690 (3010-4400)	3590 (2910-4500)	0.723
Airflow obstruction, *n* (%)	56 (16.5%)	39 (15.3%)	17 (20.0%)	0.315	38 (14.9%)	18 (21.2%)	0.180
Liver transplantation related variables							
Time since transplantation (years), median (IQR)	7.1 (2.7-13.4)	7.1 (2.8-13.3)	7.0 (2.2-13.5)	0.933	6.6 (2.1-11.9)	8.3 (5.2-15.5)	0.013
Reason for transplantation*				<0.001			0.350
- Autoimmune liver disease, *n* (%)	150 (44.1%)	126 (49.4%)	24 (28.2%)		108 (42.4%)	42 (49.4%)	
o Autoimmune hepatitis, *n* (%)	43 (12.6%)	36 (14.1%)	7 (8.2%)		33 (12.9%)	10 (11.8%)	
o Primary sclerosing cholangitis, *n* (%)	103 (30.3%)	92 (36.1%)	11 (12.9%)		72 (28.2%)	31 (36.5%)	
o Primary biliary cholangitis, *n* (%)	25 (7.4%)	14 (5.5%)	11 (12.9%)		20 (7.8%)	5 (5.9%)	
- Alcoholic or cryptogenic cirrhosis, *n* (%)	68 (20.0%)	39 (15.3%)	29 (34.1%)		47 (18.4%)	21 (24.7%)	
- Fulminant hepatic failure, *n* (%)	24 (7.1%)	15 (5.9%)	9 (10.6%)		18 (7.1%)	6 (7.1%)	
- Hepatitis C, *n* (%)	13 (3.8%)	7 (2.7%)	6 (7.1%)		9 (3.5%)	4 (4.7%)	
- Other, *n* (%)	106 (31.2%)	81 (31.8%)	25 (29.4%)		86 (33.7%)	20 (23.5%)	
Cirrhosis, any cause, *n* (%)	224 (65.9%)	164 (64.3%)	60 (70.6%)	0.355	167 (65.5%)	57 (67.1%)	0.895
Immunosuppressive medication at inclusion							
- Tacrolimus, *n* (%)	281 (82.6%)	217 (85.1%)	64 (75.3%)	0.196	216 (84.7%)	65 (76.5%)	0.036
- Ciclosporin, *n* (%)	35 (10.3%)	22 (8.6%)	13 (15.3%)		20 (7.8%)	15 (17.6%)	
- Everolimus, *n* (%)	18 (5.3%)	14 (5.5%)	4 (4.7%)		15 (5.9%)	3 (3.5%)	
- Mycophenolate mofetil, *n* (%)	247 (72.6%)	180 (70.6%)	67 (78.8%)	0.276	191 (74.9%)	56 (65.9%)	0.264
- Azathioprine, *n* (%)	31 (9.1%)	26 (10.2%)	5 (5.9%)		21 (8.2%)	10 (11.8%)	
Use of prednisolone at time of inclusion, *n* (%)	165 (48.5%)	129 (50.6%)	36 (42.4%)	0.211	125 (49.0%)	40 (47.1%)	0.803
Acute rejection, *n* (%)	41 (12.1%)	36 (14.1%)	5 (5.9%)	0.053	31 (12.2%)	10 (11.8%)	>0.99

BMI, Body Mass Index; FEV_1_, forced expiratory volume in the first second; FVC, forced vital capacity; IQR, interquartile range; kg, kilogram; m, meter; mL, milliliter.

*In some cases, more than one reason for transplantation may be listed.

### Clinical characteristics by TM and SDC-1 levels

Liver transplant recipients with elevated TM were slightly older compared to those with low TM with a median age of 60 years compared to 54 years (p<0.001). Among liver transplant recipients with elevated TM, 34.1% were never smokers, compared to 55.7% of those with low TM (p<0.01). Also, in liver transplant recipients with elevated TM, the most common reasons for transplantation were alcoholic or cryptogenic cirrhosis, whereas autoimmune liver disease was more prevalent in liver transplant recipients with low TM (p<0.001). Similarly, liver transplant recipients with elevated SDC-1 were slightly older than those with low SDC-1, with a median age of 58 years vs. 55 years (p<0.01). A higher proportion of liver transplant recipients in the group with elevated SDC-1 received cyclosporine treatment compared to those with low SDC-1 (p=0.04) (**Table 1**).

Liver transplant recipients with elevated TM had a median plasma concentration of TM of 8803 pg/mL (IQR: 7946-10093), whereas those with low TM had a median of 5097 pg/mL (IQR: 4156-5880), p<0.01. Similarly, liver transplant recipients with elevated SDC-1 had a median plasma concentration of SDC-1 of 4482 pg/mL (IQR: 4116-5236), and liver transplant recipients with low SDC-1 had a median plasma concentration of SDC-1 of 2712 pg/mL (IQR: 2189-3136), p<0.01.

### FEV_1_ and FVC by TM and SDC-1 levels

The median FEV_1_ was lower in liver transplant recipients with elevated TM than in liver transplant recipients with low TM with values of 2500 mL (IQR: 2060-3300) vs. 2850 mL (IQR: 2330-3555), p<0.01, and the median FVC was lower in liver transplant recipients with elevated TM than in liver transplant recipients with low TM with values of 3430 mL (IQR: 2690-4190) vs. 3835 mL (IQR: 3035-4455), p<0.01. In contrast, no significant differences in median FEV_1_ or FVC were observed between liver transplant recipients with elevated and low SDC-1, p=0.25 and 0.72, respectively (**Table 1**).

### Associations of TM and SDC-1 with FEV_1_ and FVC

In the linear regression analysis adjusted for age and sex, liver transplant recipients with elevated TM had 125.5 mL lower FEV_1_ than liver transplant recipients with low TM (95% CI: -274.9;23.9, p=0.101) and 163.5 mL lower FVC (95% CI: -344.2;17.2, p=0.077) (**Table 2**). When adjusted for age, sex, height, ethnicity, and smoking status, liver transplant recipients with elevated TM had 39.5 mL lower FEV_1_ than liver transplant recipients with low TM (95% CI: -173.7;94.7), p=0.564) and 72.2 mL lower FVC (95% CI: -227.2;82.8, p=0.362). Liver transplant recipients with elevated SDC-1 had 62.5 mL lower FEV_1_ than liver transplant recipients with low SDC-1 (95% CI: -210.4;85.4, p=0.408), and 1.8 mL higher FVC (95% CI: -177.5;18.1, p=0.984), when adjusted for age and sex. In the fully adjusted model, liver transplant recipients with elevated SDC-1 had 93.4 mL lower FEV_1_ than liver transplant recipients with low SDC-1 (95% CI: -223.1;36.3, 0.159) and 31.2 mL lower FVC (95% CI: -181.6;119.2, p=0.684) (**Table 2**).

**Table 2:** FEV_1_ and FVC according to thrombomodulin and syndecan-1 plasma levels.

	Thrombomodulin above 3^rd^ quartile		Thrombomodulin (log2)		Syndecan-1 above 3^rd^ quartile		Syndecan-1 (log2)	
	Estimates (95% CI)	p-value	Estimates (95% CI)	p-value	Estimates (95% CI)	p-value	Estimates (95% CI)	p-value
FEV_1_ (mL)								
Minimally adjusted*	-125.5 (-274.9;23.9)	0.101	-110.6 (-227.7;6.6)	0.065	-62.5 (-210.4;85.4)	0.408	-28.5 (-142.6;85.6)	0.625
Fully adjusted**	-39.5 (-173.7;94.7)	0.564 0.677***	-62.0 (-168.3;44.2)	0.253 0.677***	-93.4 (-223.1;36.3)	0.159 0.780***	-50.8 (-150.9;49.3)	0.321 0.780***
FVC (mL)								
Minimally adjusted*	-163.5 (-344.2;17.2)	0.077	-92.5 (-234.7;49.8)	0.203	1.8 (-177.5;181.1)	0.984	-11.1 (-149.2;127.1)	0.875
Fully adjusted**	-72.2 (-227.2;82.8)	0.362 0.677***	-46.6 (-169.5;76.3)	0.458 0.677***	-31.2 (-181.6;119.2)	0.684 0.821***	-51.3 (-167.1;64.4)	0.390 0.780***

CI, confidence interval; FEV_1_, forced expiratory volume in the first second; FVC, forced vital capacity.

*Minimally adjusted model: adjusted for age and sex.

**Fully adjusted model: adjusted for age, sex, height, ethnicity, and smoking status.

***P-values adjusted for multiple comparisons using the Benjamini–Hochberg procedure.

In addition to the dichotomized analyses, we also analyzed TM and SDC-1 as log2-transformed continuous variables to assess potential dose-dependent effects. A two-fold increase in TM corresponded with a 110.6 mL lower FEV1 (95% CI: -227.7;6.6, p=0.065), and a 92.5 mL lower FVC (95% CI: 234.7;49.8, p=0.203). Similarly, a two-fold increase in SDC-1 corresponded with a 28.5 mL lower FEV1 (95% CI: -142.6;85.6, p=0.625), and a 11.7 mL lower FVC (95% CI: -149.2;127.1, p=0.875), when adjusting for age and sex. In line with this, no statistically significant associations were found between FEV1 or FVC and log2-transformed plasma levels of TM and SDC-1 in the fully adjusted models (**Table 2**). P-values adjusted by the Benjamini-Hochberg procedure are shown in **Table 2**.

### Associations of TM and SDC-1 with airflow obstruction

The prevalence of airflow obstruction was 20.0% in liver transplant recipients with elevated TM and 15.3% in liver transplant recipients with low TM (p=0.315). In the liver transplant recipients with elevated TM, the odds ratio (OR) for airflow obstruction was 1.14 (95% CI: 0.59;2.19, p=0.693) in the minimally adjusted analysis. In the fully adjusted analysis, the results were further attenuated, with an OR of 1.01 (95% CI: 0.43;2.38, p=0.984) (**Table 3**). The prevalence of airflow obstruction was 21.2% in liver transplant recipients with elevated SDC-1 and 14.9% in liver transplant recipients with low SDC-1 (p=0.180). In liver transplant recipients with elevated SDC-1, the OR for airflow obstruction was 1.39 (95% CI: 0.73;2.64, p=0.316) in the minimally adjusted analyses. In the fully adjusted analysis, the OR was 0.97 (95% CI: 0.42;2.27, p=0.950) (**Table 3**).

**Table 3:** Odds for airflow obstruction according to thrombomodulin and syndecan-1 plasma levels.

	Thrombomodulin above 3^rd^ quartile		Thrombomodulin (log2)		Syndecan-1 above 3^rd^ quartile		Syndecan-1 (log2)	
	OR (95% CI)	p-value	OR (95% CI)	p-value	OR (95% CI)	p-value	OR (95% CI)	p-value
Airflow obstruction								
Minimally adjusted*	1.14 (0.59;2.19)	0.693	2.11 (0.95;4.67)	0.067	1.39 (0.73;2.64)	0.316	1.80 (0.83;3.90)	0.133
Fully adjusted**	1.01 (0.43;2.38)	0.984 0.984***	1.26 (0.60;2.65)	0.534 0.677***	0.97 (0.42;2.27)	0.950 0.950***	1.25 (0.62;3.53)	0.532 0.798***

CI, confidence interval; OR, odds ratio.

*Minimally adjusted model: adjusted for age and sex.

**Fully adjusted model: adjusted for age, sex, height, ethnicity, and smoking status.

***P-values adjusted for multiple comparisons using the Benjamini–Hochberg procedure.

A two-fold increase in TM levels corresponded with an OR of 2.11 (95% CI: 0.95;4.67, p=0.067) for airflow obstruction when adjusting for age and sex, while a two-fold increase in SDC-1 corresponded with an OR of 1.80 (95% CI: 0.83; 3.90, p=0.133). However, neither of the associations were statistically significant. The non-significant associations were attenuated in the fully adjusted analyses (**Table 3**). P-values adjusted by the Benjamini-Hochberg procedure are shown in **Table 3**.

Immunosuppressive medication, time since transplantation, and cirrhosis at time of transplantation were not significantly associated with airflow obstruction (data not shown).

In analyses stratified by smoking status, elevated SDC-1 was associated with higher odds for airflow obstruction with an OR of 5.59 (95% CI 1.09; 28.6, p=0.04) among liver transplant recipients who reported current smoking in the minimally adjusted model. This result was attenuated in the fully adjusted model with an OR of 4.82 (95% CI 0.92; 25.26, p=0.06). No other significant associations were found in current smokers, and no significant associations were found in never smokers (**Supplementary Tables 4** and **5**). P-values adjusted by the Benjamini-Hochberg procedure are shown in **Supplementary Tables 4** and **5**.

In exploratory analyses, sCD163 was not significantly associated with FEV_1_, FVC, or airflow obstruction in either minimally or fully adjusted models (**Supplementary Tables 6** and **7**).

Four sensitivity analyses were performed, as described in the statistics section. The four stratified analyses included: i) excluding liver transplant recipients transplanted with autoimmune liver disease, ii) excluding liver transplant recipients with acute rejection, iii) excluding liver transplant recipients receiving prednisolone treatment, and iv) only including liver transplant recipients with cirrhosis at time of transplantation. In all analyses, elevated TM or elevated SDC-1 were not significantly associated with lower FEV_1_ and lower FVC, supporting the main results. Similar consistency was observed when airflow obstruction was analyzed as the outcome.

## Discussion

In this national cross-sectional study of 340 liver transplant recipients, we found no significant associations between elevated TM or SDC-1 and FEV_1_, FVC, or airflow obstruction.

We previously reported elevated levels of F_E_NO_50_ in liver transplant recipients, raising concerns about underlying respiratory inflammation (6) and suggesting that chronic pulmonary changes may be present in liver transplant recipients. Endothelial dysfunction has previously been associated with lower FEV_1_ and FVC in healthy middle-aged women, independent of smoking status and inflammation (24). Based on these findings, we hypothesized that elevated TM or SDC-1, as markers of endothelial dysfunction, would be associated with lower FEV_1_, lower FVC, and airflow obstruction in liver transplant recipients. However, we found little evidence to support this hypothesis.

In the overall cohort, we found no significant associations between endothelial dysfunction and airflow obstruction. Several factors may help explain these findings. TM and SDC-1 may reflect early or subtle endothelial changes that precede clinically apparent respiratory disease. Previous studies have linked endothelial dysfunction to lower FEV_1_ in former smokers without COPD (25). Pulmonary endothelial abnormalities have also been observed in patients with mild COPD and in smokers with normal spirometry (26). However, TM and SDC-1 are influenced by multiple factors, including systemic inflammation, immunosuppressive medication, and vascular comorbidities (13, 27, 28). Some studies have proposed that elevated TM might reflect a compensatory upregulation of membrane-bound TM, potentially indicating a protective response rather than endothelial damage (20, 29). Elevated TM has also been associated with a reduced coronary heart disease risk (30). Similarly, low SDC-1 has been linked to reduced pulmonary function (31). These findings illustrate the complexity of interpreting these markers.

In this study, 56 of 340 liver transplant recipients met the criterion for airflow obstruction. A *post-hoc* power calculation was conducted. Assuming α = 0.05 and 80% power, with n = 85 in the elevated TM group and 255 in the low group, and a prevalence of 15.3% in the low group, a prevalence of 30.2% in the elevated group would have been required to demonstrate a statistically significant difference from 15.3% in the low group. For SDC-1, the prevalence of airflow obstruction was 21.2% in the elevated group and 14.9% in the low group. With the same assumptions (α = 0.05, 80% power, n = 85 and n = 255), a prevalence of 29.7% in the elevated group would have been required to reach statistical significance compared with 14.9% in the low group. Given these modest differences in prevalences in airflow obstruction observed between elevated and low biomarker groups, strong associations between TM or SDC-1 and airflow obstruction appear unlikely in this cohort.

In addition, our study population consisted of unselected, non-hospitalized liver transplant recipients, in contrast to previous studies that primarily included acutely ill or hospitalized patients (12–14, 32). This difference in disease severity may contribute to lower circulating TM and SDC-1 levels in our study population, potentially limiting sensitivity to detect pulmonary endothelial damage. Moreover, we measured TM and SDC-1 in plasma rather than locally. Previous studies suggest that local measurements in the lungs, such as bronchoalveolar lavage (BAL) or sputum, often better reflect pulmonary damage than systemic measurements. In one study, only TM in bronchoalveolar lavage fluid, but not in plasma, correlated with respiratory failure and lung injury severity (33). Furthermore, BAL and sputum biomarkers appear to exhibit stronger associations with lung function decline (34) and a broader spectrum of disease-related molecules (35) than plasma. This suggests that local measurements might provide greater sensitivity. Considering this, our measurements of TM and SDC-1 in plasma might reflect a similar limitation.

In other clinical settings, biomarkers are well established as tools for risk prediction and patient stratification (36). Having a similarly reliable and easily measurable biomarker for impaired lung function after liver transplantation would be valuable, as it could enable early identification of vulnerable patients and more targeted follow-up. Our findings suggest that TM and SDC-1 are not suitable for this purpose, which emphasize the need to explore alternative biomarkers that may better capture pulmonary risk in this population.

After adjusting for ethnicity and smoking status, the differences in FEV_1_ and FVC were attenuated in liver transplant recipients with elevated TM. This suggests that these factors might, at least in part, contribute to the lower FEV_1_ and FVC observed in this group. In contrast, differences became slightly more pronounced with elevated SDC-1, possibly indicating distinct underlying mechanisms. Divergent associations of TM and SDC-1 with peripheral artery disease (20) reported in a previous study support this interpretation.

Descriptive comparisons indicated that liver transplant recipients with elevated TM had lower median FEV_1_ and FVC than those with low TM, although no statistically significant associations were observed in the regression analyses. In contrast, no notable differences were observed between liver transplant recipients with elevated and low SDC-1. Some evidence suggests that the lungs are among the organs with the highest concentrations of TM (37), possibly indicating its role as a marker of pulmonary endothelial damage. While not conclusive, this may suggest that TM is more closely related to lung function than SDC-1 in this cohort.

Adjustment for immunosuppressive medication, time since transplantation, and cirrhosis at time of transplantation did not alter the associations between TM or SDC-1 and airflow obstruction. Interestingly, fewer liver transplant recipients with elevated SDC-1 were receiving tacrolimus compared to those with low levels. One study reported preserved endothelial function in tacrolimus-treated liver transplant recipients (28), suggesting that tacrolimus might influence endothelial damage markers. While our study does not allow for conclusions about causality, the observed treatment pattern might, at least in part, reflect an effect of tacrolimus on endothelial function, potentially contributing to lower TM and SDC-1 in tacrolimus-treated patients. Furthermore, both TM and SDC-1 are involved in the endothelial glycocalyx integrity and systemic inflammation (11, 12, 31), and elevated circulating levels could theoretically reflect subclinical pulmonary damage. However, our cohort consists of liver transplant recipients who receive immunosuppressive medication, which might influence systemic biomarker levels and inflammatory responses. Besides that, existing literature indicates that liver transplant recipients have an increased risk of pulmonary infections due to immunosuppressive medication (38, 39). Moreover, pulmonary infections might contribute to the mechanisms in the development of airflow obstruction (40). This emphasizes the complexity in interpreting endothelial markers in immunosuppressed individuals, because plasma measurements might not fully capture the endothelial damage.

Although we adjusted for major confounders such as age, sex, height, ethnicity, and smoking status, residual confounding cannot be ruled out. Other factors, such as cumulative smoking exposure, immunosuppressive medication, or systemic inflammation, could potentially also influence both plasma biomarker levels and pulmonary outcomes. In addition to our primary analyses, we performed additional analyses including immunosuppressive medication, time since liver transplantation, and cirrhosis at time of transplantation as covariates. These supplementary analyses yielded similar results, with no significant associations observed (data not shown), indicating that these factors did not alter the results. Additionally, in our cohort, 66% had cirrhosis at time of transplantation, but restricting our analyses to this subgroup or adjusting for cirrhosis did not notably affect the association between TM or SDC-1 and airflow obstruction. Cirrhosis is frequently observed in liver transplant candidates (1) and has been linked to pulmonary complications such as hepatopulmonary syndrome and portopulmonary hypertension (41). However, the endothelial dysfunction associated with cirrhosis (42) may be reversible following transplantation due to the resolution of portal hypertension. Therefore, cirrhosis is unlikely to influence the relationship between TM or SDC-1 and airflow obstruction in this study.

Pulmonary complications after liver transplantation are likely multifactorial, and other biological pathways, including innate immune responses and complement activation, might contribute to these outcomes. Complement activation has been implicated in acute lung injury and inflammatory lung disease, suggesting that innate immune mechanisms may be involved in pulmonary complications after liver transplantation (43). To explore one aspect of innate immunity, we analyzed sCD163, which is shed from activated monocytes and macrophages and is considered a circulating marker of innate immune activation and systemic inflammation (44). sCD163 has been associated with endothelial alterations and vascular dysfunction in various inflammatory and cardiovascular conditions (44), suggesting a link between innate immune activation and endothelial function. Despite its proposed role in systemic inflammation, we did not observe associations between sCD163 and lung function, suggesting that systemic innate immune activation, as captured by circulating sCD163, was not independently associated with lung function in this cohort.

This study has several strengths. To our knowledge, this is the first large-scale investigation of associations between TM or SDC-1 and FEV_1_, FVC, or airflow obstruction in liver transplant recipients. Moreover, the study was conducted using a large, nationwide, prospective, and well-characterized cohort, allowing for adjustment for potential confounders. Protocolized research spirometry and endothelial biomarker measurements were assessed exclusively for research purposes regardless of clinical symptoms. This approach strengthens the validity of the present findings. Additionally, protocolized research spirometry data were validated and obtained in accordance with the guidelines of ERS. However, the following limitations should be considered. As this is a cross-sectional study, neither causality nor temporal conclusions between endothelial damage and lung function can be established. Although a retrospective design might allow assessment of temporality, it would likely rely on clinically indicated spirometry and thereby increasing the risk of selection bias. Furthermore, circulating endothelial biomarkers are not part of routine clinical practice and would therefore not be systematically available in medical records. Moreover, only participants with available protocolized research spirometry were included, which may have introduced selection bias toward healthier or more compliant individuals. However, we compared baseline characteristics (age, sex, BMI, and smoking status) between participants with and without spirometry and observed no major differences in their characteristics. This reduces, but does not eliminate, the risk of selection bias. The limited number of liver transplant recipients with airflow obstruction (*n* = 56) might reduce statistical power in our analyses. And given that most liver transplant recipients were Caucasian, the results might not be generalized to other ethnic groups.

In conclusion, in this study of 340 liver transplant recipients, we did not find significant associations between elevated biomarkers of endothelial dysfunction (TM and SDC-1) and lower FEV_1_, lower FVC, or airflow obstruction. Further research is warranted to investigate the mechanism underlying the lower FEV_1_, FVC, and airflow obstruction in liver transplant recipients. Longitudinal studies of additional endothelial markers and their association with functional outcomes may further contribute to improving clinical practice.

## Data Availability

Data is available upon reasonable request and approval from primary investigator and all involved transplant centres.
