# Supplementary material for: Thrombomodulin and syndecan-1 and association with lung function in liver transplant recipients
Source: Front Immunol. 2026 Apr 13;17:1662030. doi: 10.3389/fimmu.2026.1662030 (PMC13110979; doi:10.3389/fimmu.2026.1662030)
Supplement: Supplementary file 1 [file Table4.docx]

**Supplementary**

Table 4: FEV_1_ and FVC according to thrombomodulin and syndecan-1 plasma levels stratified by smoking status

|  | Thrombomodulin  above 3^rd^ quartile | | Thrombomodulin  (log2) | | Syndecan-1  above 3^rd^ quartile | | Syndecan-1  (log2) | |
| --- | --- | --- | --- | --- | --- | --- | --- | --- |
|  | Estimate  (95% CI) | p-value | Estimate  (95% CI) | p-value | Estimate  (95% CI) | p-value | Estimate  (95% CI) | p-value |
| FEV1 in current smokers | | | | | | | | |
| Minimally adjusted* | -267.2  (-689.4;155.1) | 0.21 | -54.0  (-746.5;638.4) | 0.88 | -302.8  (-767.1;161.5) | 0.19 | -180.7  (-849.7;488.2) | 0.59 |
| Fully  adjusted** | -233.3  (-628.8;162.2) | 0.26  0.77*** | -101.4  (-538.1;335.2) | 0.65  0.82*** | -423.1  (-836.1;-10.0) | 0.05  0.26*** | -359.2  (-787.7;69.3) | 0.11  0.26*** |
| FVC in current smokers | | | | | | | | |
| Minimally adjusted* | -406.2  (-900;87.5) | 0.10 | 18.8  (-802.4;840.0) | 0.96 | -165.9  (-725.9;394.1) | 0.55 | 9.4  (-787;805.4) | 0.98 |
| Fully  adjusted** | -327.6  (-711.9;56.7) | 0.10  0.77*** | -95.9  (-528.3;336.4) | 0.67  0.82*** | -367.5  (-781.5;46.5) | 0.09  0.26*** | -387.6  (-808.8;33.7) | 0.08  0.26*** |
| FEV1 in never smokers | | | | | | | | |
| Minimally adjusted* | -18.3  (-250.0; 213.1) | 0.88 | -91.1  (-323.8; 141.5) | 0.44 | -11.0  (-201.3; 179.2) | 0.91 | 27.8  (-180.4; 236.0) | 0.79 |
| Fully  adjusted** | 18.9  (-177.9;215.7) | 0.85  0.85*** | -78.7  (-216.2;58.8) | 0.26  0.77*** | -19.4  (-180.8;142.1) | 0.82  0.82*** | 25.1  (-97.3;147.5) | 0.69  0.75*** |
| FVC in never smokers | | | | | | | | |
| Minimally adjusted* | -2.5  (-294.5; 289.6) | 0.99 | -25.5  (-319.6; 268.7) | 0.86 | 115.1  (-124.4; 354.6) | 0.34 | 81.3  (-181.2; 343.8) | 0.54 |
| Fully  adjusted** | 48.3  (-190.2;286.7) | 0.69  0.82*** | -38.9  (-206.1;128.4) | 0.65  0.82*** | 103.6  (-91.5;298.7) | 0.30  0.51*** | 64.1  (-84.0;212.2) | 0.40  0.55*** |

CI, confidence interval; FEV_1_, forced expiratory volume in the first second; FVC, forced vital capacity

*Minimally adjusted model: adjusted for age and sex

**Fully adjusted model: adjusted for age, sex, height, ethnicity, and smoking status

***P-values adjusted for multiple comparisons using the Benjamini–Hochberg procedure

Table 5: Odds for airflow obstruction according to thrombomodulin and syndecan-1 plasma levels stratified by smoking status

|  | Thrombomodulin  above 3^rd^ quartile | | Thrombomodulin  (log2) | | Syndecan-1  above 3^rd^ quartile | | Syndecan-1  (log2) | |
| --- | --- | --- | --- | --- | --- | --- | --- | --- |
|  | OR  (95% CI) | p-value | OR  (95% CI) | p-value | OR  (95% CI) | p-value | OR  (95% CI) | p-value |
| Airflow obstruction in current smokers | | | | | | | | |
| Minimally adjusted* | 1.78  (0.43;7.44) | 0.43 | 7.78  (0.50;121.75) | 0.14 | 5.59  (1.09;28.66) | 0.04 | 8.67  (0.78;96.50) | 0.08 |
| Fully  adjusted** | 1.93  (0.42; 8.93) | 0.40  0.80*** | 3.75  (0.55; 25.52) | 0.18  0.77*** | 4.82  (0.92; 25.26) | 0.06  0.26*** | 3.29  (0.60; 18.06) | 0.17  0.34*** |
| Airflow obstruction in never smokers | | | | | | | | |
| Minimally adjusted* | 0.88  (0.30;2.57) | 0.82 | 2.06  (0.65;6.56) | 0.22 | 1.55  (0.65;3.71) | 0.33 | 1.35  (0.48;3.83) | 0.57 |
| Fully  adjusted** | 0.84  (0.28; 2.47) | 0.75  0.82*** | 1.51  (0.67; 3.41) | 0.32  0.77*** | 1.45  (0.60; 3.52) | 0.41  0.55*** | 1.19  (0.57; 2.45) | 0.64  0.75*** |

CI, confidence interval; OR, odds ratio

*Minimally adjusted model: adjusted for age and sex

**Fully adjusted model: adjusted for age, sex, height, ethnicity, and smoking status

***P-values adjusted for multiple comparisons using the Benjamini–Hochberg procedure

Table 6: FEV_1_ and FVC according to CD163 plasma levels

|  | CD163  above 3^rd^ quartile | | CD163  (log2) | |
| --- | --- | --- | --- | --- |
|  | Estimates  (95% CI) | p-value | Estimates  (95% CI) | p-value |
| FEV_1_ (mL) | | | | |
| Minimally adjusted* | -106.4  (-253.4;40.7) | 0.16 | -9.6  (-80.8;61.7) | 0.79 |
| Fully adjusted** | -113.9  (-243.4;15.6) | 0.09  0.54*** | -15.4  (-78.1;47.3) | 0.63  0.73*** |
| FVC (mL) | | | | |
| Minimally adjusted* | -105.9  (-284.1;72.2) | 0.24 | -12.4  (-98.7;73.9) | 0.78 |
| Fully adjusted** | -99.3  (-249.3;50.7) | 0.20  0.60*** | -14.1  (-86.6;58.4) | 0.70  0.73*** |

CI, confidence interval; FEV_1_, forced expiratory volume in the first second; FVC, forced vital capacity

*Minimally adjusted model: adjusted for age and sex

**Fully adjusted model: adjusted for age, sex, height, ethnicity, and smoking status

***P-values adjusted for multiple comparisons using the Benjamini–Hochberg procedure

Table 7: Odds for airflow obstruction according to CD163 plasma levels

|  | CD163  above 3^rd^ quartile | | CD163  (log2) | |
| --- | --- | --- | --- | --- |
|  | OR  (95% CI) | p-value | OR  (95% CI) | p-value |
| Airflow obstruction | | | | |
| Minimally adjusted* | 1.16  (0.60;2.27) | 0.66 | 0.90  (0.64;1.25) | 0.51 |
| Fully adjusted** | 1.39  (0.69;2.82) | 0.35  0.70*** | 0.94  (0.67;1.32) | 0.73  0.73*** |

CI, confidence interval; OR, odds ratio

*Minimally adjusted model: adjusted for age and sex

**Fully adjusted model: adjusted for age, sex, height, ethnicity, and smoking status

***P-values adjusted for multiple comparisons using the Benjamini–Hochberg procedure
